# Clinical and radiographic prognostic factors in recurrent contrast-enhancing IDH-mutant gliomas treated with bevacizumab

**DOI:** 10.1093/noajnl/vdag089

**Published:** 2026-04-06

**Authors:** Collin T Le, Francesco Sanvito, Chuyin Yang, Blaine S C Eldred, Catalina Raymond, Terry J Prins, Addison Fisher, Tie Li, Linda M Liau, Robert A Chong, Phioanh Leia Nghiemphu, Timothy F Cloughesy, Albert Lai, Benjamin M Ellingson

**Affiliations:** Department of Neurology, David Geffen School of Medicine, University of California, Los Angeles, Los Angeles; UCLA Brain Tumor Imaging Laboratory (BTIL), Center for Computer Vision and Imaging Biomarkers, University of California, Los Angeles, Los Angeles; Department of Radiological Sciences, David Geffen School of Medicine, University of California, Los Angeles, Los Angeles; Department of Neurology, David Geffen School of Medicine, University of California, Los Angeles, Los Angeles; Department of Neurology, David Geffen School of Medicine, University of California, Los Angeles, Los Angeles; UCLA Brain Tumor Imaging Laboratory (BTIL), Center for Computer Vision and Imaging Biomarkers, University of California, Los Angeles, Los Angeles; Department of Radiological Sciences, David Geffen School of Medicine, University of California, Los Angeles, Los Angeles; Department of Neurology, David Geffen School of Medicine, University of California, Los Angeles, Los Angeles; Department of Neurology, David Geffen School of Medicine, University of California, Los Angeles, Los Angeles; Department of Neurosurgery, David Geffen School of Medicine, Los Angeles; Department of Neurology, David Geffen School of Medicine, University of California, Los Angeles, Los Angeles; Department of Neurology, David Geffen School of Medicine, University of California, Los Angeles, Los Angeles; Department of Neurology, David Geffen School of Medicine, University of California, Los Angeles, Los Angeles; UCLA Brain Tumor Imaging Laboratory (BTIL), Center for Computer Vision and Imaging Biomarkers, University of California, Los Angeles, Los Angeles; Department of Radiological Sciences, David Geffen School of Medicine, University of California, Los Angeles, Los Angeles; Department of Neurosurgery, David Geffen School of Medicine, Los Angeles; Department of Bioengineering, Henry Samueli School of Engineering and Applied Science, University of California, Los Angeles, Los Angeles; Department of Psychiatry, David Geffen School of Medicine at UCLA, Los Angeles

**Keywords:** bevacizumab, contrast-enhancement, glioma, IDH, RANO

## Abstract

**Background:**

While bevacizumab, an anti-angiogenic monoclonal antibody, is approved for recurrent IDH wild-type glioblastoma, reports on clinical outcomes of IDH-mutant gliomas receiving this therapy are scarce. We retrospectively evaluated prognostic variables associated with outcomes in a cohort of recurrent contrast-enhancing IDH-mutant gliomas treated with bevacizumab.

**Methods:**

Ninety-seven consecutive patients were retrospectively studied. Age, biological sex, Karnofsky performance status (KPS), number of prior recurrences, prior disease duration, 1p19q status, MGMT status, and therapy schemes were annotated as clinical variables. MRI datasets were used to quantify pre-bevacizumab contrast-enhancing tumor volume, immediate percent volumetric reduction after bevacizumab, and RANO radiographic response. Clinical and radiographic variables were evaluated as predictors of progression-free survival (PFS) and overall survival (OS) in multivariate Cox survival analyses.

**Results:**

Median PFS and OS were 3.62 and 10.49 months, respectively. A lower number of recurrences prior to bevacizumab initiation (*P *= .002 OS; *P *= .004 PFS), 1p19q codeletion (*P *= .005 OS; *P *= .0007 PFS), a higher KPS (*P *= .02 OS; *P* = ns PFS), treatment schemes including immunotherapies (*P *= .03 OS; *P* = ns PFS), a smaller baseline contrast-enhancing volume (*P *= .02 OS; *P* = ns PFS), and the achievement of RANO radiographic response (*P *= .004 OS; *P *< .0001 PFS) were independent predictors of favorable outcomes. Bevacizumab was associated with a mean corticosteroid dose reduction of 2.9 mg/day (49%) after 2 months (*P *= .001).

**Conclusion:**

This is one of the first studies to report prognostic factors associated with clinical outcomes in IDH-mutant gliomas treated with bevacizumab. Early radiographic changes may be used to monitor treatment effectiveness, and bevacizumab significantly aids the tapering of corticosteroids in this patient subset.

Key Points

- Fewer recurrences, 1p19q codeletion, and immunotherapy was favorable.
- RANO response and volumetric reduction are early MRI hallmarks of favorable outcomes.
- Corticosteroid doses for clinical management were reduced 2 months after bevacizumab initiation.

Importance of the Study

This study provides a first retrospective report of variables associated with OS and PFS in patients with recurrent contrast-enhancing IDH-mutant gliomas receiving bevacizumab treatment. Several clinical and radiographic features may be linked to clinical outcomes, including number of recurrences prior to bevacizumab initiation, 1p19q codeletion, performance status, concomitant immunotherapy, baseline contrast-enhancing tumor volume, and post-bevacizumab radiographic response. While the results of this single-arm retrospective analysis are insufficient to establish selection criteria and clinical guidelines for anti-angiogenic therapy in IDH-mutant gliomas, understanding prognostic factors and radiographic behavior associated with clinical outcomes is crucial due to the growing clinical interest for the use of bevacizumab in this specific patient population. These findings provide insights for clinical management and the formulation of hypotheses to be tested in future prospective studies. Finally, we report promising data showing early corticosteroid dose reduction following bevacizumab initiation, supporting the role of bevacizumab as a corticosteroid-sparing agent.

Gliomas, the most common primary brain cancers, comprise 81% of brain tumors in adults and remain difficult to treat, especially at recurrence.^1^ Isocitrate dehydrogenase 1/2 (IDH) mutation status is a key genetic characteristic used to inform the prognosis and treatment of glioma patients.^2-4^ IDH-mutant gliomas have their own unique characteristics, disease outcomes, and management strategies when compared to IDH wild-type glioblastoma.^5^ Bevacizumab, an antibody that binds and blocks vascular endothelial growth factor (VEGF), has become a treatment option for recurrent high grade gliomas.^6^ While multiple previous studies have focused on IDH wild-type glioblastomas,^7-10^ more recently, the TAVAREC trial evaluated bevacizumab in both IDH wild-type and IDH-mutant 1p19q-intact gliomas with histologically-proven or suspected (ie contrast-enhancing) malignant transformation at recurrence.^11^ Overall, there is a growing clinical interest in exploring the use of bevacizumab, specifically in IDH-mutant gliomas with suspected malignant transformation. In the present study, we evaluate the clinical outcomes of a large retrospective cohort of recurrent contrast-enhancing IDH-mutant gliomas treated with bevacizumab to identify favorable clinical and imaging prognostic factors that are associated with longer overall and progression-free survival.

## Methods

### Patient Cohort

In this UCLA institutional review board approved study, patients provided informed consent to allow retrospective access to their clinical, pathological, and imaging data. Patients seen at UCLA between 1998 and 2024 were screened, and all consecutive, eligible patients meeting the inclusion criteria (histopathological diagnosis of brain glioma, confirmed IDH-mutant status, bevacizumab treatment at recurrence, and available longitudinal MRI datasets before and after bevacizumab initiation) were included. Some patients received bevacizumab treatment before routine *IDH* mutational status testing (∼2009), in which case, IDH status was confirmed after the patient had already received bevacizumab. All patients also had available 1p19q codeletion status.

Clinical variables were annotated, including age at bevacizumab initiation, sex category, original, integrated histopathological and molecular diagnosis (astrocytoma vs. oligodendroglioma, as determined by 1p19q codeletion status), MGMT status, concomitant treatments, number of disease recurrences prior to bevacizumab initiation, time from original diagnosis to bevacizumab initiation, and Karnofsky performance status (KPS) at bevacizumab initiation. When available, corticosteroid (dexamethasone) doses before bevacizumab and approximately 2 months after bevacizumab initiation were annotated to determine the extent of dose reduction associated with bevacizumab initiation.

### MRI Analysis

All patients had “measurable” contrast-enhancing disease at baseline with RANO 2.0 compliant^12^ measurements. The contrast-enhancing tumor volume was quantified with segmentations, for all available MRI timepoints. For cases where T_1_w pre- and post-contrast, T_2_w, and T_2_w-FLAIR sequences were all available, the segmentation of the contrast-enhancing tumor component was performed automatically with NS-HGlio artificial intelligence tool (Neosoma Inc, Groton, MA, United States, https://neosomainc.com).^13^ For cases with missing sequences, the contrast-enhancing tumor component was segmented semi-automatically by manually contouring an approximate tumor region, and then applying intensity thresholding on T_1_w subtraction maps, as previously described,^14^ to isolate only contrast-enhancing tumor regions without cystic or necrotic components. The semi-automated segmentations, as well as the quality control and edits to the automated segmentations, were conducted by a trained lab member (C.T.E.) under the supervision of a neuroradiologist with 8 years of experience in neuroimaging research (F.S.).

### Overall Survival and Progression-Free Survival Determination

OS was defined as the time from bevacizumab initiation to patient death or last date of clinical contact (OS, Supplementary Figure S1A). Time to Bevacizumab represented the duration of disease prior to bevacizumab treatment and was calculated as the time from initial surgery to bevacizumab initiation (Supplementary Figure S1A). Progression-free survival (PFS, Supplementary Figure S1A) was calculated from the start of bevacizumab to the day of progressive disease (PD), according to a rigorous retrospective re-assessment of the date of PD using the RANO 2.0 criteria without the use of confirmation scans.^12^ Quantitative measures of contrast-enhancing tumor volume from the segmentations were input into the Automated Imaging Response Evaluation System (AIRES) software.^15^ Progressive disease (PD) was defined as either a ≥ 40% enlargement of the contrast enhancing tumor volume (as identified by AIRES) *or* as an unequivocal non-enhancing progression on T_2_w images (retrospectively reviewed by a neuroradiologist, F.S.). Unequivocal non-enhancing progression was evaluated qualitatively and defined as a clear extension of the T_2_/FLAIR hyperintense tumor, with clear features suggesting tumor growth, such as mass effect or evident cortical/gray matter infiltration. T_2_/FLAIR alterations ascribable to vasogenic edema, leukoencephalopathy, or macroangiopathic modifications were disregarded. This approach is compliant with RANO 2.0 guidelines for contrast-enhancing tumors treated with anti-angiogenic agents, for which PD can correspond to a quantitative contrast-enhancing progression and/or a qualitative non-enhancing progression.^12^ RANO radiographic response was defined as a tumor showing greater than 65% reduction in contrast-enhancement (CE) volume for two scans with ≥4 week separation after bevacizumab initiation. Improvement in non-enhancing tumor extent was not considered response.^16^

### Statistical Analyses

To evaluate the association between variables and PFS/OS, univariate and multivariate survival analyses were conducted using Cox proportional hazards regression. The following covariates were used in the Cox analyses: age at bevacizumab initiation, sex category, time to bevacizumab from diagnosis, KPS at bevacizumab initiation (categorized in ≤70 vs. >70), MGMT-promoter methylation status, treatment scheme (bevacizumab monotherapy vs + cytotoxic therapy vs + immunotherapy vs + IDH inhibitor), the number of recurrences prior to bevacizumab initiation (as a continuous variable), original histopathological diagnosis (oligodendroglioma IDH-mutant 1p19q-codeleted vs astrocytoma IDH-mutant 1p19q-intact), baseline CE volume at the time of bevacizumab initiation, immediate bevacizumab-induced percent CE volume reduction, and RANO radiographic response. The abovementioned multivariate Cox model was used to evaluate the association of each clinical and imaging variable with PFS and OS, while adjusting for all other covariates included in the model. Additionally, Kaplan–Meier (KM) curves were generated for selected covariates to visualize the survival differences between patient groups, and log-rank tests (or log-rank for trend, in the case of ordered variables) were used to show group differences along with these plots. Of note, the KM depiction required categorization of continuous variables (eg number of recurrences prior to bevacizumab initiation was analyzed as a continuous variable in Cox models and grouped as 1 vs. 2 vs. 3+ for log-rank). Pre- and post-bevacizumab steroid doses (when available) were compared with paired Wilcoxon sign-ranked tests. Changes in corticosteroid dose were reported as both a percent change and change in mg/day dose. Differences in RANO response rates (Fisher’s exact tests) and percent CE volume reduction (Mann-Whitney *U* tests) between histopathological groups and recurrence groups were also tested. All statistical tests and survival analyses were conducted in GraphPad Prism (Boston, MA, United States).

## Results

### Cohort Characteristics

We identified 97 patients who met the selection criteria (diagnosis of IDH-mutant glioma (*n* = 94 with IDH1 mutation and *n* = 3 with IDH2 mutation), bevacizumab treatment at recurrence, and available longitudinal MRI datasets (descriptive characteristics in Table 1)). All patients had measurable contrast-enhancing tumors at the time of bevacizumab initiation. The pre-bevacizumab MRI used as baseline was acquired 10.2 (mean) ± 13.5 (standard deviation) days prior to bevacizumab initiation. Median PFS and OS were 3.6 and 10.5 months, respectively, in this cohort (Supplementary Figure S1C-D). 51 patients experienced contrast-enhancing progression, 32 had non-enhancing progression, and 14 were censored for PFS. Median PFS was 2.7 months for the non-enhancing progression group and 3.8 months for the contrast-enhancing progression group (*P *= .1), while the median OS were 9.5 and 10.3 months, respectively (*P *= .5).

**Table 1.** Cohort Characteristics.

		All (*n* = 97) (%)	1^st^ rec. (*n* = 16) (%)	2^nd^ rec.(*n* = 20) (%)	≥3^rd^ rec.(*n* = 61) (%)
Mean age at Bevacizumab initiation (years)		41.87	43.13	40.09	42.13
Sex (Male)		71 (73)	9 (56)	16 (80)	46 (75)
MGMT promoter					
	Methylated	35 (36)	7 (44)	6 (30)	22 (36)
	Unmethylated	20 (21)	2 (13)	7 (35)	11 (18)
	Unknown	42 (43)	7 (44)	7 (35)	28 (46)
Mean KPS at Bevacizumab initiation		81.03	78.13	86.00	80.16
	>70	74 (76)	11 (69)	19 (95)	44 (72)
	<=70	23 (24)	5 (31)	1 (5)	17 (28)
Treatment scheme					
	Monotherapy	30 (31)	4 (25)	6 (30)	20 (33)
	Combined therapy	67 (69)	12 (75)	14 (70)	41 (67)
	+ Cytotoxic Therapy	46 (47)	10 (63)	8 (40)	28 (46)
	+ Immunotherapy	10 (10)	1 (6)	2 (10)	7 (11)
	+ IDH Inhibitor	11 (11)	1 (6)	4 (20)	6 (10)
Histopathological diagnosis					
	Oligodendroglioma	26 (27)	3 (19)	3 (15)	20 (33)
	Astrocytoma	71 (73)	13 (81)	17 (85)	41 (67)

Demographic and clinical characteristics of 97 patients with IDH-mutant recurrent gliomas, at the time of bevacizumab initiation.

Abbreviations: Bev, bevacizumab; IDH, isocitrate dehydrogenase; KPS, Karnofsky performance status; rec, recurrence; MGMT, O6-methylguanine-DNA methyltransferase.

#### Histopathology and Tumor Grade

The original tumor diagnoses were oligodendroglioma (IDH-mutant 1p19q-codeleted, *n* = 26) or astrocytoma (IDH-mutant 1p19q-intact, *n* = 71). For 58 patients, lesions already had a histopathological grade of 3 or 4 at the time of initial diagnosis. For 29 patients, lesions initially had a histological grade of 2 at first surgery but received an updated grading of 3 or 4 following surgical re-sampling (with re-resection or biopsy) at one of the recurrences prior to bevacizumab initiation. For the remaining 10 patients, it was not possible to demonstrate a histological malignant transformation to grade 3 or 4 because tissue was not collected at the time of bevacizumab initiation. However, it is reasonable to assume that the remaining 10 lesions had also transformed to grade 3 or 4, given that all patients had measurable contrast-enhancing disease according to RANO 2.0 criteria, which is a radiographic feature strongly associated with malignant transformation on histopathology.^17^

#### Rationale for Bevacizumab Initiation

In most cases (*n* = 81 patients), bevacizumab was initiated due to suspected tumor progression (Supplementary Table S1). Among these, 11 patients had previously experienced chemotherapy-induced neutropenia/thrombocytopenia; therefore, therapeutic alternatives were particularly limited. In other patients, bevacizumab was started as a medical treatment for suspected radiation necrosis (*n* = 4), to reduce blood-brain barrier permeability^18^ and control mass effect from peritumoral edema (*n* = 6), or with the intent of tapering steroids (*n* = 4).

#### Treatment Schemes

Bevacizumab was administered in monotherapy (*n* = 30) or combined with other therapies (*n* = 67) (Table 1), including cytotoxic therapies (*n* = 46, mainly chemotherapy), immunotherapies (*n* = 10, mainly immune checkpoint inhibitors), or IDH inhibitors (*n* = 10, almost exclusively ivosidenib) (Supplementary Table S2).

#### Prior Treatments

Patients were treated with bevacizumab at either first recurrence (*n* = 16 patients), second recurrence (*n* = 20), or at third or later recurrence (*n* = 61). All patients had received concurrent chemoradiation with temozolomide prior to bevacizumab initiation. The median time from radiation completion to bevacizumab initiation was 15.0 months (13.4 oligodendrogliomas vs 16.0 astrocytomas) (8.6 1^st^ recurrence, 20.6 2^nd^ recurrence, 20.1 months 3^rd^+ recurrences), and this time interval was ≤3 months for a minority of patients (*n* = 4 oligodendrogliomas, *n* = 11 astrocytomas). Several patients had also received other chemotherapies, immunotherapies, or IDH inhibitors (Supplementary Table S3) prior to bevacizumab.

#### History of Disease

Time to bevacizumab was used as a measure of the duration of the disease from diagnosis to bevacizumab initiation (Supplementary Figure S1A). Time to bevacizumab was shorter in astrocytomas (median: 52.2 months) compared to oligodendrogliomas (median: 92.0 months) (*P *= .003) (Supplementary Figure S1B).

### A Lesser Number of Recurrences Predicts Longer PFS and OS under Bevacizumab

**Figure 1.**
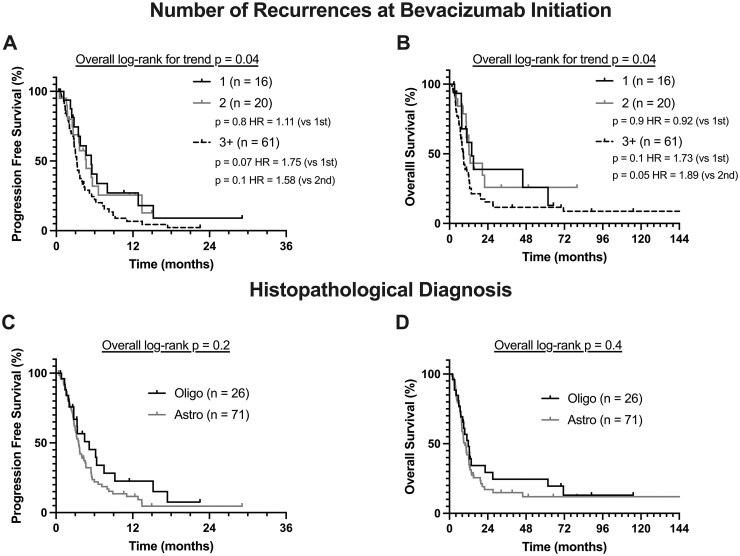
Association of clinical variables with PFS and OS. Kaplan-Meier curves representing the impact of (A and B) number of recurrences at Bevacizumab initiation (1, 2, or 3+) and (C and D) histopathological diagnosis on PFS and OS. Pairwise comparisons were conducted with log-rank test for trend (number of recurrences) and log-rank test (diagnosis).

A lesser number of recurrences was a significant predictor of longer PFS (*P *= .004, HR = 1.35) and OS (*P *= .002, HR = 1.41) in multivariate Cox analyses when adjusting for all other covariates, including age and time to bevacizumab (Table 2). Since the number of recurrences was used as a continuous variable in these models, these HR values can be interpreted as each additional recurrence being associated with a 35% and 41% increased hazard of PFS and OS, respectively. Similar results were seen in univariate analysis (Supplementary Table S4). When plotting KM curves for one, two, or more than three recurrences prior to starting bevacizumab, median PFS was 5.5, 4.7, and 3.2 months, and OS was 13.9, 12.2, and 8.7 months, respectively (Figure 1A and B). The corresponding KM curves and statistical analysis (*P *= .04) confirm an increasingly worse prognosis with an increasing number of prior recurrences (Figure 1A and B).

**Table 2.** Multivariate Cox survival analysis of PFS and OS under bevacizumab treatment.

Variables	PFS		OS	
	HR (95% CI)	*P* value	HR (95% CI)	*P* value
Age at Bev initiation	0.997 (0.97-1.03)	.8	0.998 (0.97-1.02)	.8
Time to Bevacizumab	1.00 (0.994-1.01)	.8	1.00 (0.997-1.01)	.3
Sex (*F* = ref)	1.25 (0.71-2.30)	.5	0.88 (0.50-1.63)	.7
KPS				
>70	1.0 (ref)		1.0 (ref)	
≤70	0.82 (0.41-1.56)	.6	2.10 (1.11-3.88)	.02*
MGMT methylation	1.97 (0.85-4.72)	.1	1.25 (0.58-2.79)	.6
Treatment scheme				
Bevacizumab Monotherapy	1.0 (ref)		1.0 (ref)	
Bevacizumab + Cytotoxic Therapy	1.41 (0.69-2.95)	.4	1.47 (0.76-2.91)	.3
Bevacizumab + Immunotherapy	2.93 (0.86-8.57)	.06	0.32 (0.10-0.87)	.03*
Bevacizumab + IDH Inhibitor	1.45 (0.58-3.53)	.4	1.55 (0.55-3.98)	.4
Number of recurrences at initiation	1.35 (1.10-1.65)	.004*	1.41 (1.14-1.76)	.002*
Histopathological diagnosis				
Oligodendroglioma	1.0 (ref)		1.0 (ref)	
Astrocytoma	3.67 (1.77-8.03)	.0007*	2.61 (1.35-5.22)	.005*
Baseline CE volume	1.01 (0.99-1.03)	.2	1.02 (1.002-1.03)	.02*
% CE volume reduction	0.9995 (0.997-1.003)	.8	0.997 (0.994-1.002)	.2
RANO radiographic response	0.10 (0.05-0.20)	<.0001*	0.43 (0.24-0.76)	.004*

Abbreviations: Bev, bevacizumab; CE, contrast-enhancing; IDH, isocitrate dehydrogenase; KPS, Karnofsky performance status; MGMT, O6-methylguanine-DNA methyltransferase; RANO, response assessment in neuro-oncology.

*p < 0.05

### Astrocytoma Histopathology Was Associated with Shorter PFS and OS

In multivariate Cox analysis adjusting for other prognostic factors (Table 2), astrocytomas 1p19q-intact had significantly shorter PFS (*P *= .0007, HR = 3.67) and OS (*P *= .005, HR = 2.61) compared to oligodendrogliomas 1p19q-codeleted (which was used as a reference class of the categorical variable). The KM survival curves for these histopathological groups is useful to visualize this difference (Figure 1C and D), although it is not statistically significant in log-rank or univariate analysis not adjusting for covariates.

### Other Clinical Predictors of PFS and OS

A worse performance status (KPS ≤70) at bevacizumab initiation (*P *= .02, HR = 2.10) was an independent predictor of shorter OS from bevacizumab initiation (but not of PFS) in multivariate analyses adjusting for other variables. Treatment schemes that included concurrent immunotherapy treatment in addition to bevacizumab were associated with longer OS (*P *= .03, HR = 0.32) but not significantly associated with PFS (Table 2). Other clinical variables showed some association with OS and PFS in univariate analysis (Supplementary Table S4), but not after adjusting for covariates.

### Imaging Predictors of PFS and OS

At the first post-bevacizumab MRI follow-up (mean interval = 1.4 months), the mean CE volume reduction was –57.1%. 39 patients (40%) achieved RANO radiographic response on bevacizumab treatment (≥65% volumetric reduction maintained ≥4 weeks). The RANO response rate did not significantly differ between histopathological groups or recurrence groups, nor did the percent of volumetric reduction (Supplementary Figure S2).

**Figure 2.**
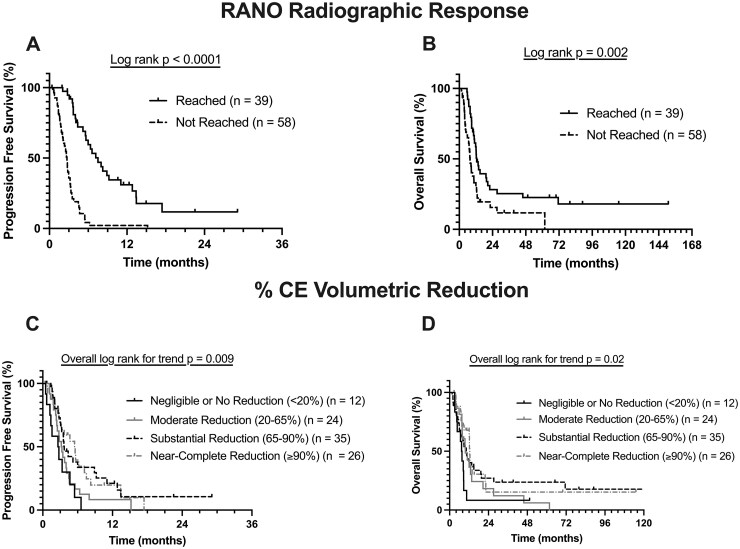
Association of imaging-derived variables with PFS and OS. Kaplan–Meier curves representing the impact of (A and B) RANO radiographic response status and (C and D) % CE volumetric reduction at post-bevacizumab follow-up on PFS and OS. Patients were categorized as having negligible or no reduction (<20%), moderate reduction (20%-65%), substantial reduction (65%-90%), or near-complete reduction (≥90%).

**Figure 3.**
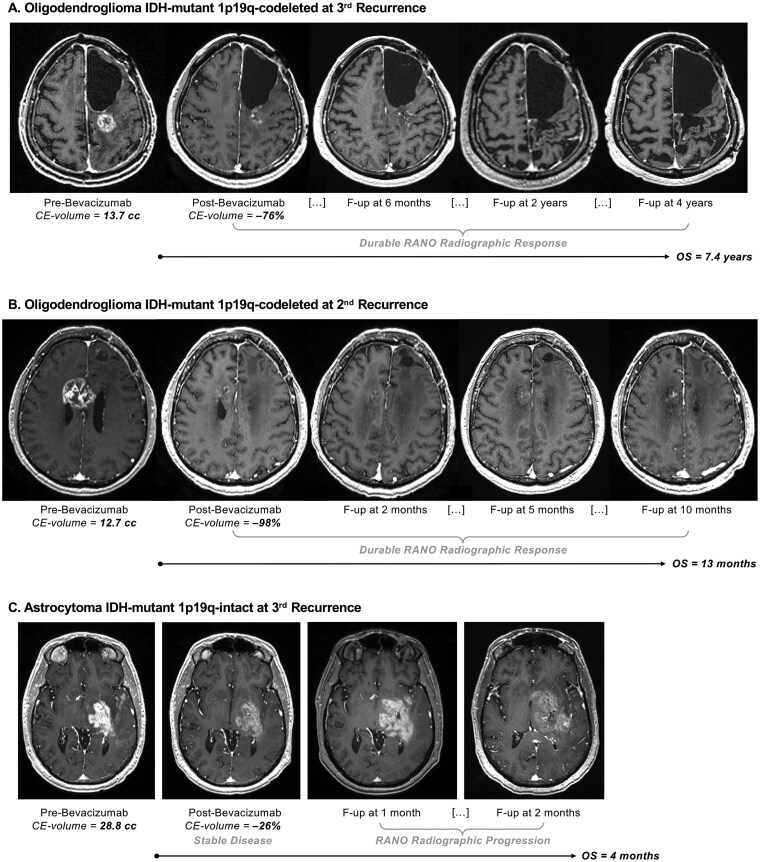
Representative cases of post-bevacizumab radiographic response patterns. Patients (A and B) with a smaller pre-bevacizumab CE tumor volume, deeper post-bevacizumab tumor shrinkage, and RANO durable radiographic response tend to have longer OS compared to patients (C) without these favorable imaging findings. For each patient, images were selected to display the slice with the largest tumor area at each time point.

RANO radiographic response was a strong, independent predictor of both favorable PFS (*P *< .0001, HR = 0.10) and OS (*P *= .004, HR = 0.43) under bevacizumab treatment in multivariate survival analysis adjusting for all other clinical and radiographic covariates (Table 2). The visualization of KM curves shows how patients that reached RANO radiographic response had improved PFS (*P *< .0001) and OS (*P *= .002) (Figure 2A and B). A greater percent reduction of CE volume was a predictor of prolonged PFS and OS only in univariate analysis (Supplementary Table S4). Percent reduction in CE volume and RANO radiographic response demonstrated collinearity, with a median CE volume reduction of 83% in the RANO response group versus 59.3% in the non-response group (*P *= .002). For data visualization with KM curves, the percentage of immediate CE tumor volumetric reduction was categorized as follows: near-complete reduction of the CE tumor component (≥90% volumetric reduction), substantial reduction (65%-90% reduction, where 65% corresponds to the threshold for a RANO-defined response or preliminary response), moderate reduction (20%-65%), negligible or no reduction (<20% reduction or enlargement). This categorization is arbitrary and only serves data visualization purposes, while the formal statistical analysis was conducted with Cox models using the volumetric reduction percentage as a continuous variable. The KM curve visualization shows proportionality between the percent volumetric reduction and PFS (*P *= .009) and OS (*P *= .02) (Figure 2C and D). Figure 3 shows representative cases with different radiographic patterns following bevacizumab.

A smaller baseline pre-bevacizumab CE volume was an independent predictor of improved OS (but not PFS) in multivariate and univariate analysis (Table 2, Supplementary Table S4).

### Sensitivity Analyses of the Multivariate Model

We conducted two additional sensitivity analyses to address potential limitations of the original multivariate modeling. Because bevacizumab was initiated with different rationale across the cohort, we conducted a sensitivity analysis by re-running the multivariate model only in the patient subset (*n* = 81) that received bevacizumab due to suspected tumor recurrence (Supplementary Table S5). Additionally, because the original multivariate model slightly exceeded the commonly recommended ratio of 10 events per covariate, we repeated the analysis including only the covariates that were significant in the original multivariate model (Supplementary Table S6). These analyses confirmed the robustness of the main findings, with oligodendroglioma histopathology, fewer prior recurrences, and achievement of a RANO radiographic response remaining independent, favorable predictors of both PFS and OS.

### Reduction in Daily Corticosteroid Dose after 2 Months of Bevacizumab Treatment

**Figure 4.**
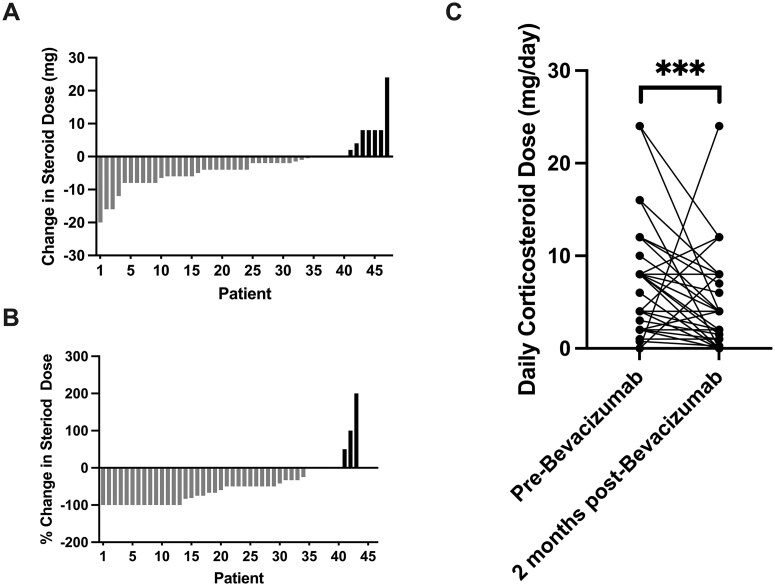
Corticosteroid dose reductions following bevacizumab initiation. (A) Change in mg/day corticosteroid dose and (B) % change in corticosteroid dose between Bevacizumab initiation and 2 months post-Bevacizumab initiation. Gray indicates a reduction in corticosteroid dose, and black indicates an increase. (C) Scatter dot plot showing daily corticosteroid (dexamethasone) dose at the time of bevacizumab initiation and 2 months after initiation.

Corticosteroid doses at bevacizumab initiation and ∼2 months after initiation were available from medical records in 48 patients. In this sub-cohort, the median time of the post-bevacizumab corticosteroid dose assessment was 1.7 months. Of 48 patients, 44 were receiving corticosteroids at bevacizumab initiation (mean dose: 7.6 mg/day), while 4 were off corticosteroids. Of those on corticosteroids, 35 (80%) experienced a dose reduction, including 14 (32%) who discontinued entirely, 6 (14%) maintained an unchanged dose, and 3 (7%) required a dose increase. All 4 patients initially not taking corticosteroids required an increase in corticosteroid dose by 2 months due to early radiographic progression with worsened vasogenic edema. While these four patients represented an exception, there was an average corticosteroid dose decrease of 2.9 mg/day and a mean percent reduction of 49% in this sub-cohort, overall. Bevacizumab treatment was associated with a significant reduction in daily corticosteroid dose (*P *= .001) (Figure 4).

## Discussion

Bevacizumab is an anti-angiogenic treatment that is part of the clinical management of brain gliomas. While it is typically administered to patients with glioblastoma IDH wild-type, it is recently also being used in patients with IDH-mutant glioma at recurrence, in the presence of histologically-proven or radiographically-suspected malignant transformation. In our single-institution, retrospective study of 97 IDH-mutant recurrent contrast-enhancing gliomas, we examined the utilization of bevacizumab in IDH-mutant gliomas and evaluated prognostic clinical and radiographic features associated with PFS and OS, including number of recurrences prior to bevacizumab initiation, concomitant treatments, and molecular profiles.

In our cohort, patients with fewer recurrences prior to bevacizumab initiation had better outcomes when treated with bevacizumab. However, it is important to note that this effect is not due to longer prior history of disease (and thus shorter residual survival), since disease duration was explicitly accounted for in our analyses. Additionally, this finding should not necessarily be interpreted as *earlier* bevacizumab use in IDH-mutant will result in a survival advantage, as this has not been shown to be the case in IDH wild-type gliomas, where deferred administration had similar outcomes compared to administration at earlier recurrences.^7^ One possible explanation for our finding is that fewer recurrences likely represent a favorable prognostic factor regardless of therapy, as lesions with more recurrences are more likely to have transformed into higher grades and/or may represent more aggressive tumors, independent from the prior duration of disease. Data from a matched control cohort without bevacizumab treatment is needed to disentangle these effects.

Comparisons between bevacizumab treatment schemes in multivariate analysis revealed prolonged OS for patients receiving bevacizumab plus immunotherapy, compared to bevacizumab alone. This finding may suggest that the addition of immunotherapy to bevacizumab can provide a survival benefit, but it may also be influenced by underlying biological features of the small subset of the tumors selected for immunotherapy (ie a selection bias), although our analyses control for the main prognostic factors such as age, contrast-enhancing tumor volume, and disease duration. The possibility of a selection bias would be in line with previous evidence from a randomized phase II clinical trial on IDH wild-type glioblastoma, which showed no synergistic benefit of immunotherapy plus bevacizumab compared to immunotherapy alone.^19^ The interaction between bevacizumab and immunotherapies are of particular interest due to the possibility of using bevacizumab as an alternative to corticosteroids that should not interfere with immunotherapies.

As for imaging-derived variables, the achievement of a RANO radiographic response was a strong and independent favorable predictor of both PFS and OS in this cohort of IDH-mutant gliomas. The association between RANO radiographic response and OS found here may appear surprising given the historical lack of association between objective response rates and OS across clinical trials employing antiangiogenic treatments in glioblastoma.^20^ This discrepancy may be partially linked to the adjustments for all clinical and imaging-derived variables performed in the present study. Additionally, a link between the volumetric reduction of the contrast-enhancing component and OS has been seen in studies adopting contrast-enhancing T1_w_ subtraction maps to more accurately quantify the contrast-enhancing volumes before and after initiation of anti-angiogenic agents.^21^^,^^22^ Similar to these studies, our results are also based on an accurate quantification of contrast-enhancing volumes as we used an automated AI tool that accounts for both pre- and post-contrast T1_w_ images and/or T1_w_ subtraction maps for semi-manual segmentations.

The immediate percent volumetric reduction after bevacizumab treatment was also a predictor of PFS and OS in univariate analysis. The demonstrated collinearity between percent reduction of CE volume and the achievement of RANO radiographic response reasonably explains why only one of the two emerged as a significant *independent* predictor of PFS/OS in multivariate analysis. Overall, our findings suggest that the degree of reduction in the contrast enhancing tumor and whether a significant (≥65%) volumetric reduction is maintained for ≥4 weeks could represent early imaging findings that support bevacizumab effectiveness in IDH-mutant gliomas.

Our results from an early assessment of corticosteroid doses ∼2 months after bevacizumab initiation also showed that bevacizumab can help reduce the corticosteroid dose needed for clinical management. Bevacizumab has started to be increasingly employed as a “super-steroid” and has been shown to improve clinical symptoms and reduce vasogenic edema.^23^ Our finding that bevacizumab treatment is associated with daily corticosteroid dose reduction supports the use of bevacizumab as a steroid-sparing agent.^24^ This is especially relevant due to the well-known adverse effects of long-term corticosteroid use. These results highlight the use of bevacizumab not only in disease control but in improving patient quality of life through decreased corticosteroid dependence.

### Limitations

The single-arm and retrospective nature of the study did not allow us to disentangle whether the variables associated with clinical outcomes are predictive factors specific to bevacizumab-treated IDH-mutant gliomas or more generic prognostic factors applicable to any treatment regimen. Analyzing the association between concomitant treatments (eg immunotherapy) and outcomes was inevitably linked to potential selection bias for these treatments, due to the retrospective design of the analysis. This retrospective design also resulted in certain incomplete data. Corticosteroid information was rather sparse and did not allow a thorough assessment of the steroid-sparing role of bevacizumab beyond the earlier timepoints following treatment initiation. Additionally, an updated histopathological diagnosis with tumor grade at the time of bevacizumab initiation was not available for all cases, although a high-grade lesion was proven in the majority of cases (*n* = 87) and reasonably suspected in the remaining cases (*n* = 10). This cohort was heterogeneous both in terms of disease history and rationale for bevacizumab initiation. However, the multivariate analysis accounted for objective metrics reflecting disease history and patient status such as time to bevacizumab, number of prior recurrences, and KPS. The sensitivity analysis also confirmed that the main results held true specifically in the subset of patients with suspected tumor growth. A minority of these patients (*n* = 15) had completed radiation ≤3 months before bevacizumab; therefore, we cannot rule out that the suspected progression might be pseudoprogression, which is more frequent in this time interval.

## Conclusion

In this IDH-mutant glioma cohort, a lower number of recurrences prior to bevacizumab initiation, 1p19q codeletion, concomitant immunotherapies, and RANO radiographic response were associated with improved PFS and OS. These findings represent an initial descriptive report of prognostic factors associated with clinical outcomes under bevacizumab treatment in contrast-enhancing, recurrent IDH-mutant glioma patients and highlight that early imaging findings may serve as potential indicators of treatment benefit. Additionally, bevacizumab showed promise as a corticosteroid-sparing agent in these patients, supporting its potential use as a corticosteroid alternative.

## Supplementary Material

vdag089_Supplementary_Data

## Data Availability

Data from this cohort is available from the authors upon request.
