# Supplementary material for: Clinical and radiographic prognostic factors in recurrent contrast-enhancing IDH-mutant gliomas treated with bevacizumab
Source: Neurooncol Adv. 2026 Apr 6;8(1):vdag089. doi: 10.1093/noajnl/vdag089 (PMC13110796; doi:10.1093/noajnl/vdag089)
Supplement: vdag089_Supplementary_Data [file vdag089_supplementary_data.zip › NOA-D-25-00601R1_Supplements_Clean.docx]

**Clinical and radiographic prognostic factors in recurrent contrast-enhancing *IDH*-Mutant Gliomas treated with Bevacizumab**

**Supplementary Material**

**
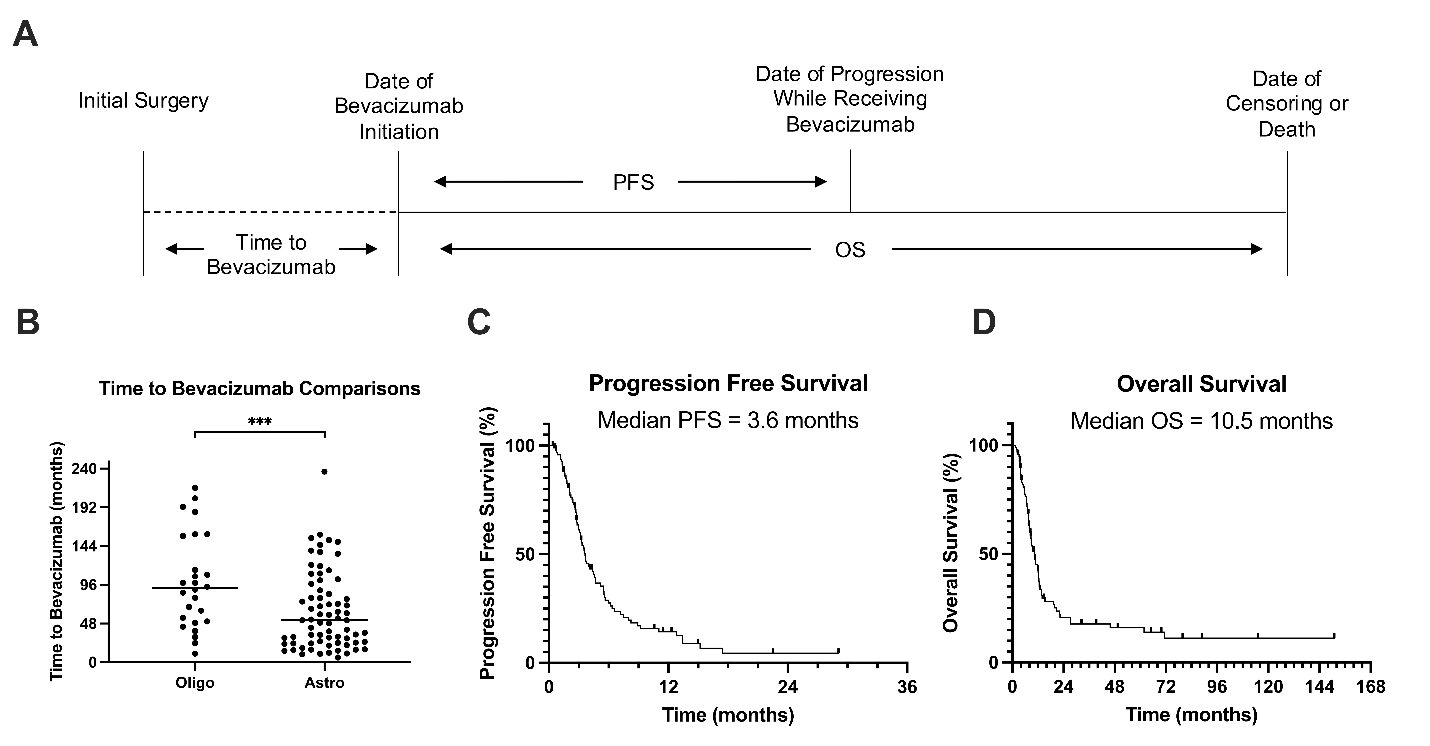
**

**Figure S1: Descriptive statistics of time to bevacizumab, PFS, and OS.**

**(A)** Schematic diagram defining survival intervals. Time to Bevacizumab: time between initial surgery and date of bevacizumab initiation; PFS: time between date of bevacizumab initiation and date of tumor recurrence after bevacizumab initiation; OS: time between date of bevacizumab initiation and date of death or censor. **(B)** Comparison of time to bevacizumab in patients with oligodendroglioma 1p19q-codeleted vs astrocytoma 1p19q-intact. **(C-D)** KM curves of PFS and OS for the full cohort.

| **Table S1: Documented Clinical Rationale for Bevacizumab Initiation** | | | | |
| --- | --- | --- | --- | --- |
|  | All (n=97) | 1^st^ rec. (n = 16) | 2^nd^ rec. (n = 20) | 3^rd^+ rec. (n = 61) |
| Suspected tumor progression | 81 | 9 | 17 | 55 |
| subset with previous chemotherapy-induced toxicity | 11 | 1 | 2 | 8 |
| Suspected radiation necrosis | 4 | 3 | 0 | 1 |
| Worsened edema without suspicion for progression | 6 | 2 | 1 | 3 |
| Steroid-tapering without suspicion for progression | 4 | 2 | 0 | 2 |
| Clinical rationale not available | 2 | 0 | 2 | 0 |

| **Table S2: Concomitant treatments administered concomitant to Bevacizumab** | | | | | |
| --- | --- | --- | --- | --- | --- |
|  |  | All (n=97) (%) | 1^st^ rec. (n = 16) (%) | 2^nd^ rec. (n = 20) (%) | 3^rd^+ rec. (n = 61) (%) |
| Treatment Scheme | |  |  |  |  |
|  | Combined | 67 (69) | 12 (75) | 14 (70) | 41 (67) |
|  | Monotherapy | 30 (31) | 4 (25) | 6 (30) | 20 (33) |
| Combination Schemes | |  |  |  |  |
|  | +Irinotecan | 16 | 3 | 2 | 11 |
|  | +Lomustine | 12 | 4 | 4 | 4 |
|  | +Ivosidenib | 10 | 1 | 4 | 5 |
|  | +Carboplatin | 10 | 0 | 1 | 9 |
|  | +Pembrolizumab | 8 | 0 | 2 | 6 |
|  | +Temozolomide | 5 | 3 | 1 | 1 |
|  | +Etoposide | 2 | 0 | 0 | 2 |
|  | +Radiation | 3 | 1 | 0 | 2 |
|  | +Enasidenib | 1 | 0 | 0 | 1 |
|  | +Dendritic Cell Vaccine | 1 | 1 | 0 | 0 |
|  | +Toca 511 | 1 | 0 | 0 | 1 |

| **Table S3: Treatments administered prior to Bevacizumab** | | | | |
| --- | --- | --- | --- | --- |
|  | All (n=97) | 1^st^ rec. (n = 16) | 2^nd^ rec. (n = 20) | 3^rd^+ rec. (n = 61) |
| Radiation | 97 | 16 | 20 | 61 |
| Temozolomide | 97 | 16 | 20 | 61 |
| Lomustine | 48 | 3 | 8 | 37 |
| Carboplatin | 22 | 0 | 0 | 22 |
| PCV | 12 | 1 | 3 | 8 |
| Ivosidenib | 10 | 1 | 3 | 6 |
| Pembrolizumab | 10 | 1 | 2 | 7 |
| Etoposide | 8 | 0 | 0 | 8 |
| Dendritic Cell Therapy | 5 | 1 | 1 | 3 |
| Optune | 5 | 2 | 0 | 3 |
| Accutane | 5 | 0 | 0 | 5 |
| Vorasidenib | 4 | 1 | 0 | 3 |
| Irinotecan | 2 | 0 | 0 | 2 |
| Enasidenib | 2 | 0 | 0 | 2 |
| Toca-511 | 2 | 0 | 0 | 2 |
| Cabozantinib | 2 | 0 | 0 | 2 |
| Vincristine | 2 | 0 | 0 | 2 |
| Imatinib | 2 | 0 | 0 | 2 |
| Other* | 14 | 0 | 0 | 14 |

*Other: AEE788-RAD001, Antineoplaston therapy, CAR T, Carmustine, Erlotinib, GDC-0084, Iressa, Paclitaxel, Pexidartinib, Poly-ICLC, Sorafenib, Tamoxifen, Thalidomide, Voxtalisib.

| **Table S4. Univariate Cox Survival Analysis of PFS and OS under Bevacizumab Treatment** | | | | |
| --- | --- | --- | --- | --- |
| Factors | PFS | | OS | |
|  | HR (95% CI) | *p* value | HR (95% CI) | *p* value |
| Age at Bev Initiation | 0.98 (0.96 – 1.01) | 0.1 | 0.99 (0.97 – 1.02) | 0.5 |
| Time to Bevacizumab | 1.00 (0.996 – 1.004) | 0.99 | 1.00 (0.997 – 1.01) | 0.7 |
| Sex (F = ref) | 0.98 (0.61 – 1.62) | 0.9 | 0.78 (0.47 – 1.34) | 0.3 |
| KPS |  |  |  |  |
| >70 | 1.0 (ref) |  | 1.0 (ref) |  |
| ≤70 | 0.84 (0.47 – 1.43) | 0.5 | 1.45 (0.84 – 2.42) | 0.2 |
| MGMT Methylation | 0.76 (0.40 – 1.49) | 0.4 | 1.03 (0.53 – 2.09) | 0.9 |
| Treatment Scheme |  |  |  |  |
| Bevacizumab Monotherapy | 1.0 (ref) |  | 1.0 (ref) |  |
| Bevacizumab + Cytotoxic Therapy | 1.23 (0.73 – 2.16) | 0.5 | 1.57 (0.91 – 2.79) | 0.1 |
| Bevacizumab + Immunotherapy | 1.25 (0.41 – 3.11) | 0.7 | 0.97 (0.35 – 2.28) | 0.9 |
| Bevacizumab + IDH Inhibitor | 2.14 (0.98 – 4.46) | 0.05* | 1.64 (0.64 – 3.76) | 0.3 |
| Number of Recurrences at Initiation | 1.22 (1.05 – 1.40) | 0.009* | 1.24 (1.06 – 1.44) | 0.006* |
| Histopathological Diagnosis |  |  |  |  |
| Oligo | 1.0 (ref) |  | 1.0 (ref) |  |
| Astro | 1.46 (0.87 – 2.57) | 0.2 | 1.26 (0.76 – 2.16) | 0.4 |
| Baseline CE Volume | 1.004 (0.99 – 1.02) | 0.6 | 1.02 (1.001 – 1.03) | 0.03* |
| % CE Volumetric Reduction | 0.997 (0.995 – 1.00) | 0.02* | 0.996 (0.994 – 0.9995) | 0.008* |
| RANO Radiographic Response | 0.19 (0.11 – 0.31) | <0.0001* | 0.47 (0.28 – 0.76) | 0.002* |

**Figure S2: Association between radiographic response, histopathological diagnosis, and number of prior recurrences.**

Comparisons of RANO radiographic response status and percent CE volumetric reduction for patients stratified by **(A-B)** histopathological diagnosis (oligodendroglioma vs astrocytoma) and **(C-D)** number of recurrences at bevacizumab initiation (1, 2, or 3+). This analysis revealed no statistical difference (*p*>0.05) for all these comparisons.

| **Table S5. Sensitivity Analysis: Multivariate Cox Survival Analysis of PFS and OS  (subset of n=81 patients with suspected tumor progression at bevacizumab initiation)** | | | | |
| --- | --- | --- | --- | --- |
| Factors | PFS | | OS | |
|  | HR (95% CI) | *p* value | HR (95% CI) | *p* value |
| Age at Bev Initiation | 0.996 (0.96 – 1.03) | 0.8 | 1.00 (0.97 – 1.03) | 0.998 |
| Time to Bevacizumab | 0.998  (0.991 – 1.01) | 0.6 | 0.9997  (0.993 – 1.01) | 0.9 |
| Sex (F = ref) | 1.39 (0.74 – 2.75) | 0.3 | 0.9 (0.49 – 1.73) | 0.7 |
| KPS |  |  |  |  |
| >70 | 1.0 (ref) |  | 1.0 (ref) |  |
| ≤70 | 0.76 (0.36 – 1.51) | 0.4 | 1.70 (0.86 – 3.25) | 0.1 |
| MGMT Methylation | 2.44 (0.88 – 7.06) | 0.09 | 1.92 (0.76 – 5.09) | 0.2 |
| Treatment Scheme |  |  |  |  |
| Bevacizumab Monotherapy | 1.0 (ref) |  | 1.0 (ref) |  |
| Bevacizumab + Cytotoxic Therapy | 1.50 (0.66 – 3.64) | 0.4 | 1.45 (0.71 – 3.04) | 0.3 |
| Bevacizumab + Immunotherapy | 4.70 (1.32 – 14.87) | 0.01* | 0.47 (0.13 – 1.44) | 0.2 |
| Bevacizumab + IDH Inhibitor | 0.96 (0.31 – 2.84) | 0.9 | 1.77 (0.54 – 5.34) | 0.3 |
| Number of Recurrences at Initiation | 1.36 (1.08 – 1.71) | 0.008* | 1.55 (1.23 – 1.95) | 0.0002* |
| Histopathological Diagnosis |  |  |  |  |
| Oligodendroglioma | 1.0 (ref) |  | 1.0 (ref) |  |
| Astrocytoma | 4.94 (2.19 – 11.94) | 0.0002* | 3.24 (1.58 – 6.90) | 0.002* |
| Baseline CE Volume | 1.01 (0.99 – 1.03) | 0.6 | 1.01 (0.996 – 1.03) | 0.1 |
| % CE Volume Reduction | 0.9995  (0.996 – 1.003) | 0.7 | 0.998  (0.994 – 1.002) | 0.3 |
| RANO Radiographic Response | 0.08 (0.03 – 0.20) | <0.0001* | 0.42 (0.22 – 0.81) | 0.009* |

| **Table S6. Sensitivity Analysis: Multivariate Cox Survival Analysis of PFS and OS  (using only significant covariates from Table 2)** | | |
| --- | --- | --- |
| **Factors** | **PFS** | |
|  | HR (95% CI) | *p* value |
| Number of Recurrences at Initiation | 1.35 (1.16 – 1.57) | 0.0001* |
| Histopathological Diagnosis |  |  |
| Oligodendroglioma | 1.0 (ref) |  |
| Astrocytoma | 3.11 (1.67 – 6.15) | 0.0006* |
| RANO Radiographic Response | 0.14 (0.08 – 0.25) | <0.0001* |
| **Factors** | **OS** | |
|  | HR (95% CI) | *p* value |
| KPS |  |  |
| >70 | 1.0 (ref) |  |
| ≤70 | 2.02 (1.12 – 3.51) | 0.02* |
| Treatment Scheme |  |  |
| Bevacizumab Monotherapy | 1.0 (ref) |  |
| Bevacizumab + Cytotoxic Therapy | 1.83 (1.05 – 3.31) | 0.04* |
| Bevacizumab + Immunotherapy | 0.37 (0.12 – 0.94) | 0.049* |
| Bevacizumab + IDH Inhibitor | 1.40 (0.53 – 3.39) | 0.5 |
| Number of Recurrences at Initiation | 1.47 (1.22 – 1.79) | <0.0001* |
| Histopathological Diagnosis |  |  |
| Oligodendroglioma | 1.0 (ref) |  |
| Astrocytoma | 2.23 (1.21 – 4.25) | 0.01* |
| Baseline CE Volume | 1.02 (0.99 – 1.03) | 0.04* |
| RANO Radiographic Response | 0.39 (0.22 – 0.65) | 0.0004* |
