# Supplementary figures and images for: Clinical and radiographic prognostic factors in recurrent contrast-enhancing IDH-mutant gliomas treated with bevacizumab
Source: Neurooncol Adv. 2026 Apr 6;8(1):vdag089. doi: 10.1093/noajnl/vdag089 (PMC13110796; doi:10.1093/noajnl/vdag089)

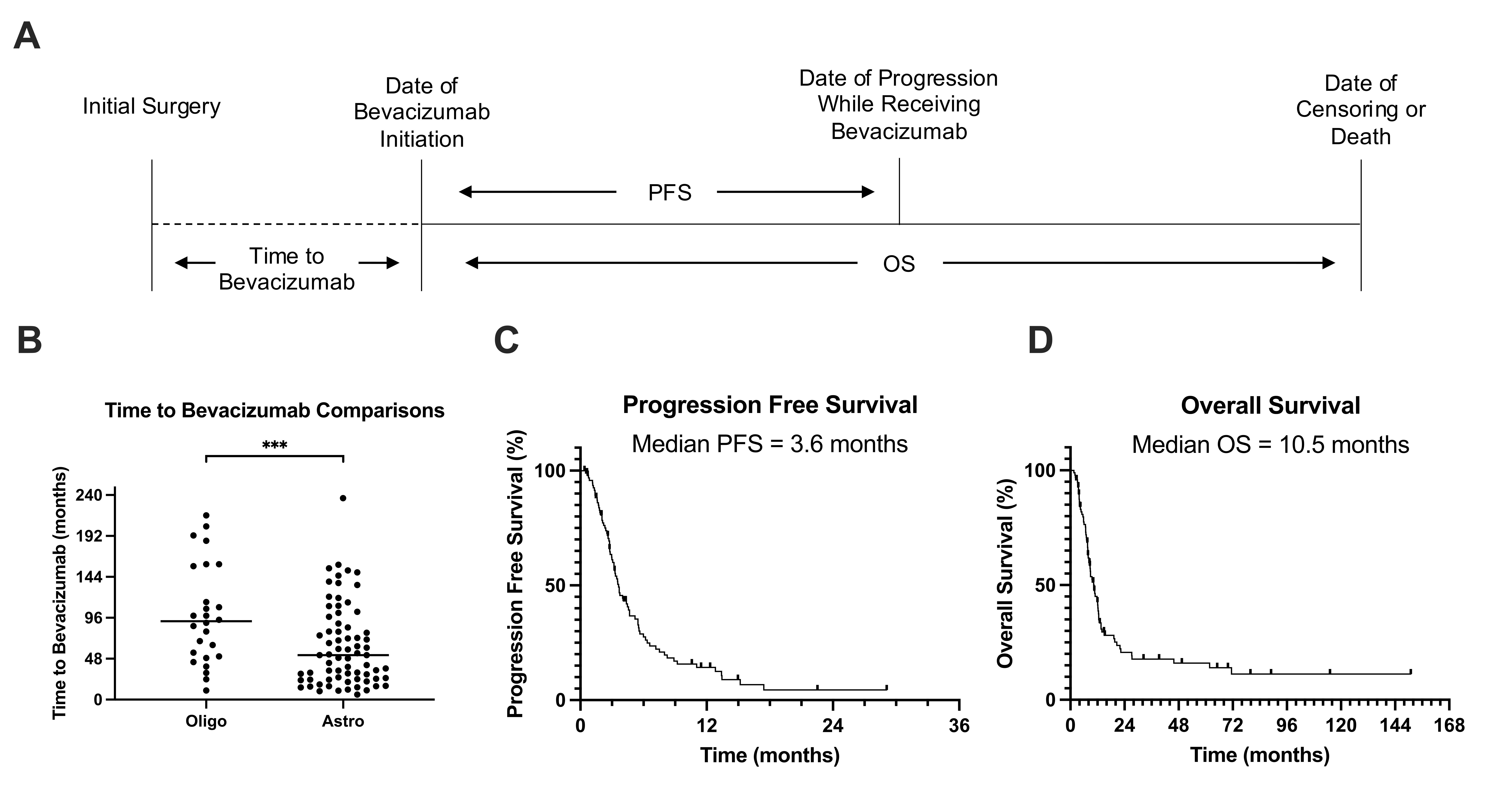

Supplement: vdag089_Supplementary_Data [file vdag089_supplementary_data.zip › Supplementary Figure S1.tif]
